# Dietary calcium intake in Brazilian preschoolers and schoolchhildren: review of the literature

**DOI:** 10.1590/1984-0462/2023/41/2021253

**Published:** 2022-07-06

**Authors:** Rodrigo André Galvão, Bruna Pavon, Maria Carolina Brandão Morán, Maria Victória Costa Barbin, Ana Luiza Cabrera Martimbianco, Guido de Paula Colares

**Affiliations:** a Centro Universitário São Camilo, São Paulo, SP, Brazil.

**Keywords:** Calcium, dietary, Child, Brazil, Dairy products, Cálcio na dieta, Criança, Brasil, Laticínios

## Abstract

**Objective:**

The objective of this study was to map and synthesize evidence on the adequacy of dietary calcium intake and dairy products in Brazilian preschoolers and schoolchildren.

**Data source:**

Evidence searches were performed in the MEDLINE (via PubMed) and Latin American and Caribbean Health Sciences Literature (LILACS; via BVS) databases, with no restriction on date or language of publication. Experimental or observational studies that evaluated healthy Brazilian children between 2 and 12 incomplete years old were included.

**Data synthesis:**

A total of 18 studies were included. Seven of 11 studies of 11 studies (63.6%) identified mean values of dietary calcium intake below the age recommendation, especially in schoolchildren, with the progression of the age group. Among preschoolers, studies with direct weighing of food showed higher mean values of dietary calcium ingested compared to those with dietary recall. Children attending public daycare centers on a part-time basis tended to have inadequate calcium intake. The consumption of milk and dairy products was lower among older children, especially schoolchildren.

**Conclusions:**

Inadequate dietary calcium intake seems to be prevalent in Brazil during childhood, especially among schoolchildren. Therefore, the evaluation of milk and dairy products intake must be considered in order to desgn appropriate corrective actions.

## RESUMO

**Objetivo:**

Mapear e sintetizar as evidências sobre a adequação do consumo de cálcio dietético e laticínios em crianças brasileiras pré-escolares e escolares.

**Fontes de dados:**

As buscas pelas evidências foram realizadas nas bases de dados *Medical Literature Analysis and Retrieval System Online* (Medline, via PubMed) e Literatura Latino-Americana e do Caribe em Ciências da Saúde (Lilacs, via Biblioteca Virtual em Saúde — BVS), sem restrição de data ou idioma de publicação. Foram incluídos estudos experimentais ou observacionais que avaliaram crianças brasileiras saudáveis com idade entre dois e 12 anos incompletos.

**Síntese dos dados:**

Foram incluídos 18 estudos. Sete de 11 estudos (63,6%) identificaram valores médios da ingesta de cálcio dietético abaixo do recomendado para a idade, principalmente em escolares, com a progressão da faixa etária. Entre os pré-escolares, estudos com pesagem direta dos alimentos apresentaram maiores valores médios de cálcio dietético ingerido comparados aos obtidos com recordatório alimentar. Crianças frequentadoras de creches públicas em regime de meio período tiveram a maior inadequação da ingesta de cálcio. A ingesta de leite e derivados foi menor entre as crianças com idade mais avançada, principalmente em escolares.

**Conclusões:**

A inadequação da ingesta de cálcio dietético parece ser prevalente no Brasil, principalmente em escolares. Sendo assim, a avaliação da ingestão de leite e derivados é um ponto a ser observado para a realização de ações corretivas nessa faixa etária.

## INTRODUCTION

Calcium is one of the minerals responsible for bone formation and mineralization when synthesizing hydroxyapatite crystals.^1^ In childhood, adequate calcium intake is essential for bone accretion during skeletal growth and the range of bone mass peak suitable for prevention of osteoporosis and fractures in adulthood.^2^ In addition, the intake of elemental calcium in the diet negatively correlates with cardiovascular disease by helping to reduce blood pressure through the renin-angiotensin-aldosterone system, decreasing fat absorption, and increasing lipolysis with lower lipid concentrations and body weight consequently.^3,4^

Dietary calcium is mainly present in milk and dairy products, but foods, such as sardines, spinach, cabbage, and broccoli, are sources with a lower mineral concentration in daily diet. After ingestion, approximately 20–60% of dietary calcium is absorbed in the duodenum and jejunum, stimulated by calcitriol, the biologically active form of vitamin D. In addition, periods of physiological change, such as growth in childhood, puberty, pregnancy, and lactation, increase the percentage of calcium absorption to maintain adequate bone mineralization.^1^

During childhood, the daily recommendation of dietary calcium increases gradually with age. According to the Sociedade Brasileira de Pediatria, infants in the first year of life should receive 200–260mg of dietary calcium. In contrast, preschoolers who aged between 2 and 6 years incomplete, and schoolchildren who aged between 7 and 12 years incomplete, as stated by the Estatuto da Criança e do Adolescente (ECA), should ingest approximately 700–1300mg of calcium. Despite this, in Brazil, according to the Pesquisa de Orçamentos Familiares 2017–2018, household consumption of dairy products decreased by 36% compared to 2002–2003, mainly fresh products.^5,6^

In the first 2 years of life, milk intake predominates in the infants feeding, which guarantees the daily needs of calcium generally, but dairy consumption tends to decrease with the introduction of other types of food in the following years.^7^ Therefore, this study aimed to evaluate the adequacy of dietary calcium intake in Brazilian preschoolers and schoolchildren.

## METHOD

A synthesis of evidence was performed through a narrative review, which either considered eligible experimental and observational comparative studies or not (i.e., cohort, case-control, and cross-sectional), which performed up to the time of the search, and which assessed the adequacy of the daily consumption of dietary calcium and milk and dairy products in healthy Brazilian children who aged between 2 and 12 years incomplete. Reports and case series, foreign studies, and articles including patients with preexisting comorbidities, such as lactose intolerance and cow's milk allergy (CMA), were excluded.

In September 2020, the search for evidence was conducted in MEDLINE database via PubMed – National Library of Medicine of the National Institutes of Health and Latin American and Caribbean Health Sciences Literature (LILACS) database via the Virtual Health Library (VHL). Chart 1 presents the search strategies elaborated for each database.

**Chart 1:** Search strategies for each database.

MEDLINE (via PubMed)	#1 “Calcium, Dietary”[Mesh] OR (Dietary Calcium) OR (Calcium intake) #2 “Dairy Products”[Mesh] OR (Dairy Product) OR (Product, Dairy) OR (Products, Dairy) #3 #1 OR #2 #4 “Child”[Mesh] OR Children #5 “Brazil”[Mesh] #6 #3 AND #4 AND #5
LILACS (via VHL)	#1 MH:(Cálcio na Dieta) OR (Calcium, Dietary) OR (Calcio en la Dieta) OR D01.146.395 OR MH: Laticínios OR (Dairy Products) OR (Productos Lácteos) OR G07.203.300.350 OR J02.500.350 #2 MH: Criança OR Child OR Niño OR M01.060.406 #3 MH: Brasil OR Brazil OR Brasil OR Z01.107.757.176 #4 #1 AND #2 AND #3

Two independent authors selected the studies obtained from the search using the Rayyan platform, a free web and mobile application, developed by the Qatar Computing Research Institute (QCRI) as an auxiliary tool for archiving, organizing, and selecting articles using a process of semi-automation.^8^ The first stage of the study selection was performed through the analysis of titles and abstracts. The studies considered potentially eligible were then evaluated in full text to verify the eligibility criteria by both independent authors. Finally, disagreements were resolved by a third reviewer, in consensus with the previous ones.

The data from the included studies were extracted and synthesized narratively, considering the following characteristics: the state where the study was performed, study design, sample size, age and gender of the participants, the instrument for evaluating outcomes (i.e., dietary recall, food frequency questionnaire, or direct weighing of food), values of calcium intake in milligrams (mg), and the percentage of the adequacy of daily intake of calcium or milk and dairy products intake in the diet.

## RESULTS

**Figure 1:**
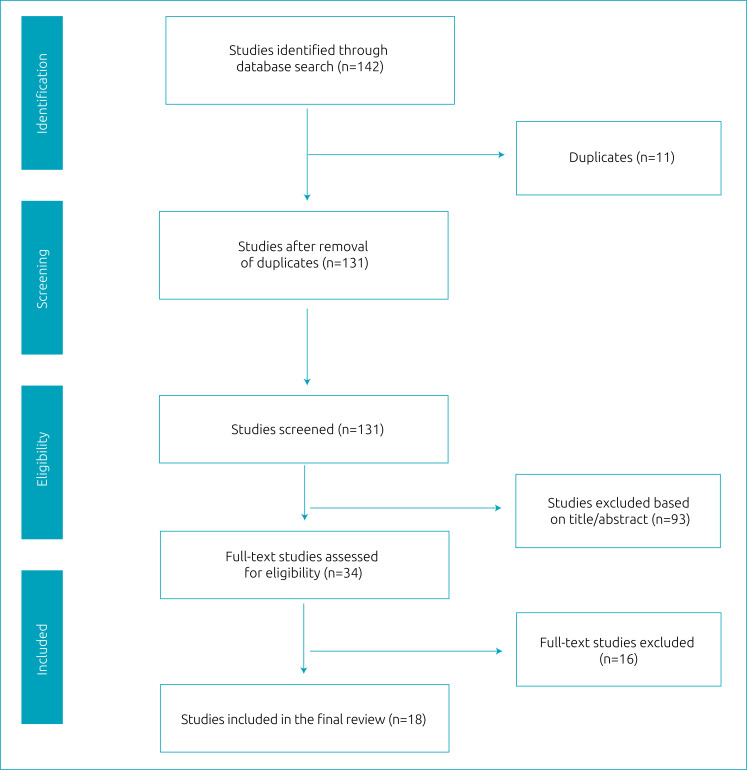
Flowchart of the study selection process.

The search in the databases resulted in 142 articles, of which 34 were selected for full reading after removal of duplicates and irrelevant articles. After selection, 18 manuscripts were considered eligible and selected to write this article (Figure 1).

The methodological characteristics of the selected studies, the types of intervention, and the results obtained are detailed in Tables 1–4. Nine studies evaluated dietary calcium intake, seven estimated milk and dairy products consumption, and two performed both evaluations.

**Table 1:** Identification and methodological characteristics of the selected studies for dietary calcium intake evaluation.

Author	State	Study type	Sample n (M/F)	Age range (y)	Location	Instrument
Nascimento et al.^17^	Ceará	Cohort	40 (17/23)	7–11.9	HH	Dietary recall
Martino et al.^10^	Minas Gerais	CS	151 (82/69)	4–6	DCC	Weighted diet record
Scagliusi et al.^16^	Acre	CS	61 (28/33)	6–9	HH	24-h dietary recall/food-frequency questionnaire
Tavares et al.^11^	Amazonas	CS	308 (161/147)	2–3	DCC/HH	DCC: weighted diet record HH: dietary recall
Bernardi et al.^12^	Rio Grande do Sul	CS	362 (166/196)	2–6	DCC/HH	DCC: weighted diet record HH: dietary recall
Bueno et al.^13^	Multicentre	CS	3,058 (1,571/1,487)	2–6	DCC/HH	DCC: weighted diet record HH: food diary
Castro et al.^27^	Multicentre	CS	2266 (1,158/1,108)	3–6	DCC/HH	DCC: weighted diet record HH: dietary recall
Magalhães et al.^18^	Minas Gerais	CS	347 (142/205)	8–9	HH	Dietary recall
Alencar et al.^14^	Piauí	CS	81 (42/39)	4–5	DCC	Food dietary record/weighted diet record
Suhett et al.^15^	Minas Gerais	CS	350 (166/184)	8–9	HH	24-h dietary recalls
Leroux et al.^9^	São Paulo	CS	64 (34/30)	1–4	HH	24-h duplicate dietary recall

n: number, M: male, F: female, y: year, CS: cross-sectional, DCC: daycare center, HH: household.

**Table 2:** Results obtained from the selected studies regarding dietary calcium intake.

Author	Sample n (M/F)	Instrument	Dietary calcium reference range (mg)	Dietary calcium intake (mg)	Inadequate daily calcium intake % (n)
Nascimento et al.^17^	40 (17/23)	Dietary recall	7–11.9 y: 1,100	466.8±293.6	97.5 (39)
Martino et al.^10^	151 (82/69)	Weighted diet record	4–6 y: 1,000	182.4 [132.9; 229.0]	100
Scagliusi et al.^16^	61 (28/33)	24-h dietary recall/food-frequency questionnaire	6–8 y: 1,000 9 y: 1,300	252.5	NA
Tavares et al.^11^	308 (161/147)	DCC: weighted diet record HH: dietary recall	2–3 y: 700	Public: 885.0±17.6 Private: 920.0±26.3	Public: 27.6 (60) Private: 7.9 (7) Total: 21.8 (67)
Bernardi et al.^12^	362 (166/196)	DCC: weighted diet record HH: dietary recall	2–3 y: 700 4–6 y: 1,000	2–3y: 852.6±246.9 4–6y: 808.4±250.8	Public: 32.2 (83) Private: 33.7 (35) Total: 32.6 (118)
Bueno et al.^13^	3,058 (1,571/1,487)	DCC: weighted diet record HH: food diary	2–3 y: 500 4–6 y: 800	2–3 y Public: 821.6 Private:762.2 4–6 y Public: 804.1 Private: 792.3	2–3 y Public: 12.6 Private:13.6 4–6 y Public: 48.9 Private: 40.3
Castro et al.^27^	2,266 (1,158/1,108)	DCC: weighted diet record HH: dietary recall	3 y: 700 4–6 y: 1,000	728.4±354.2	NA
Magalhães et al.^18^	347 (142/205)	Dietary recall	8 y: 1,000 9 y: 1,300	458.4	NA
Alencar et al.^14^	81 (42/39)	Food dietary record/weighted diet record	4–5 y: 1,000	PT:34.8±23.8 −62.3±49.1 FT: 371.1±142.1	PT: 61.1–78.2 FT: 33.7
Suhett et al.^15^	350 (166/184)	24-h dietary recalls	NA	NA	97.4 (341)
Leroux et al.^9^	64 (34/30)	24-h duplicate dietary recall	1–3 y: 700 4 y: 1,000	1–3 y: 500.8±207.3 4 y: 461.3±223.5	1–3 y: 50.0 (22) 4 y: 93.3 (18)

n: number, M: male, F: female, y: year, DCC: daycare center, HH: household; NA: not available, PT: part-time, FT: full-time, mean±standard deviation, median [minimum, maximum].

**Table 3:** Identification and methodological characteristics of the selected studies for the intake of milk and dairy products evaluation.

Author	State	Study type	Sample n (M/F)	Age range (y)	Location	Instrument
Rauber et al.^21^	Rio Grande do Sul	Cohort	276 (NA)	3–4; 7–8	HH	Dietary recall
Pretto et al.^24^	Rio Grande do Sul	Cohort	616 (316/300)	8	HH	Food Guide Based Questionnaire
Bielemann et al.^22^	Rio Grande do Sul	Cohort	3,316 (1,723/1,593)	4–6	HH	Dietary recall
Levy-Costa et al.^25^	São Paulo	CS	204 (NA)	2–5	HH	Dietary recall
Bernardi et al.^12^	Rio Grande do Sul	CS	362 (166/196)	2–6	DCC/HH	DCC: weighted diet record HH: dietary recall
Oliveira S. Filha et al.^19^	Sergipe	CS	65 (35/30)	2.0–2.9	HH	Dietary recall
Bortolini et al.^20^	Multicenter	CS	2,732 (NA)	2.0–4.9	HH	Dietary recall
Martins et al.^23^	Ceará	CS	200 (NA)	3–5	HH	Food Frequency Questionnaire
Suhett et al.^15^	Minas Gerais	CS	350 (166/184)	8–9	HH	Dietary recall

n: number, M: male, F: female, y: year, CS: cross-sectional, NA: not available, DCC: daycare center, HH: household.

**Table 4:** Results obtained from the selected studies regarding the intake of milk and dairy products.

Author	Sample, n (M/F)	Instrument	Inadequate milk and dairy products intake, % (n)	Percentage of total daily calories derived from milk (%)
Rauber et al.^21^	276 (NA)	Dietary recall	3–4 y Intervention: 23.4 (34) Control: 30.5 (61) 7–8 y Intervention: 57.3 (75) Control: 56.2 (99)	NA
Pretto et al.^24^	616 (316/300)	Food Guide-Based Questionnaire	75 (462)	NA
Bielemann et al.^22^	3,316 (1,723/1,593)	Dietary recall	4 y: M: 43.5 (749) F: 47.3 (753) 6 y: M: 51.5 (831) F: 59.3 (899)	NA
Levy-Costa et al.^25^	204 (NA)	Dietary recall	NA	24–35 mo: 21.8 36–47 mo: 20.6 48–60 mo: 13.1
Bernardi et al.^12^	362 (166/196)	DCC: weighted diet record HH: dietary recall	13.2 (45)	NA
Oliveira S. Filha et al.^19^	65 (35/30)	Dietary recall	55.4 (36)	NA
Bortolini et al.^20^	2,732 (NA)	Dietary recall	55.2 (1508)	NA
Martins et al.^23^	200 (NA)	Food Frequency Questionnaire	Milk: 14.5 (29) Dairy products: 60.5 (121)	NA
Suhett et al.^15^	350 (166/184)	Dietary recall	34 (119)	NA

n: number, M: male, F: female, y: year, CS: cross-sectional, NA: not available, DCC: daycare center, HH: household, mo: months old.

Among the 11 studies that evaluated dietary calcium intake by different methods, such as dietary recalls and weighted dietary records, 7 studies identified mean values below the recommended amount for age (Tables 1 and 2). Leroux et al.^9^ found a higher percentage of inadequate calcium consumption among 4-year-old preschoolers (93.3%) compared to children who aged 1–3 years (50%). Similarly, Martino et al.^10^ found lower dietary calcium intake values in all study children who aged 4–6 years.

By directly weighing the food offered in daycare centers and recalling the diet offered at home, Tavares et al.^11^ and Bernardi et al.^12^ found a lower prevalence of inadequate dietary calcium intake in preschoolers. In contrast, Bueno et al.^13^ identified lower dietary calcium consumption among preschoolers who aged 4–6 years than younger children.

When evaluating the daycare center and school schedule, Tavares et al.^11^ concluded that children attending private daycare centers have a higher prevalence of calcium intake than public daycare students. Besides, Alencar et al.^14^ identified a more significant inadequacy of dietary calcium consumption among part-time preschoolers (61.1–78.2%) compared to full-time students (33.7%).

Among preschoolers, studies involving the direct weighing of foods showed a higher mean of dietary calcium values ingested than those performed with dietary recall.^11–13^

In children who aged older than 6 years, dietary calcium intake progressively decreases with advancing age. All four studies with dietary recall identified low calcium consumption with values equivalent to half recommended for age.^15–18^

Regarding the prevalence of adequate milk and dairy products intake, equivalent to three daily shares, Oliveira S. Filha et al.^19^ and Bortolini et al.^20^ found percentages below 50% among preschoolers.^5^ In addition, Rauber et al.^21^ found higher milk and dairy products consumption among preschoolers than schoolchildren in both control and intervention groups (Tables 3 and 4).

There is a reduction in the percentage of milk and dairy products intake with advancing age. Bielemann et al.^22^ noticed a higher consumption of dairy products in 4-year-old children than preschoolers who aged 6 years, with no significant difference between the sex. Bernardi et al.^12^ found low values of inadequate dairy intake (13.4%) in preschoolers, and Martins et al.^23^ identified inadequate milk intake (14.5%) in the same age group. In contrast, Pretto et al.^24^ Levy-Costa et al.^25^ identified 75% of 8-year-old children without the recommended consumption. corroborated this finding by identifying a progressive decrease in the percentage of milk and dairy products in the diet of preschoolers according to age.

## DISCUSSION

Most of the studies evaluated in this review indicate a lower calcium intake in Brazilian preschoolers and schoolchildren because the identified values of dietary calcium and milk and dairy consumption were below the values recommended for each age group by the Sociedade Brasileira de Pediatria.^5^

Due to this fact, a diet rich in calcium should be stimulated in these age groups once this mineral is fundamental, during the child's longitudinal growth, for adequate bone formation and mineralization and consequent reduction of skeletal comorbidities, such as osteoporosis and fractures, in adulthood.^26–28^

The deficiency in calcium intake among children is associated with eating habits, ethnic and geographical differences, and cultural, socioeconomic, and lifestyle factors.^29^ Given these factors, the performance of studies evaluated in different Brazilian states, whose population has peculiarities in culture, food, and socioeconomic status, may have contributed to the variations found in the evaluation of calcium consumption in preschoolers and schoolchildren.

Moreover, the research instrument may have contributed to the differences in dietary calcium intake results in preschoolers. The highest values were obtained in the studies with the individual direct weighing of food, a more objective measure, than studies with a dietary recall. Dietary recalls and food diaries may not reflect the usual diet due to bias like the dependence on the memory of the interviewed person, alterations made by the patient to attend to the interviewer's expectations, and difficulties in food portion estimation.^30^

Some studies of this review, such as the study by Tavares et al.,^11^ Bernardi et al.,^12^ and Rauber et al.,^21^ have shown a higher calcium intake in children who aged up to 4 years. During infancy, the encouragement of breastfeeding to reduce the chance of early weaning and the use of infant formulae may influence the consumption of milk and dairy products at the beginning of the preschool phase.^31^

However, there is a progressive increase in the inadequacy of dairy and dietary calcium consumption with the advancement of chronological age, especially in those older than 6 years. In this age group, socioeconomic conditions, the school food environment, parental food choices, dietary patterns, a greater consumption of soft drinks, industrialized juices, and other ultraprocessed foods are essential factors influencing these outcomes.^6,32^ Moreover, more restrictive diets and food selectivity may have influenced this result. Vegetarian diets have become more common in various age groups due to health events and religious or philosophical principles. They can lead to calcium deficiency because they may contain large amounts of fibers, phytates, and oxalates, compounds capable of chelating calcium and reducing its passive absorption in the distal jejunum and ileum.^33–36^

Cow's milk is a potentially allergenic food, triggering atopic reactions in infants predisposed to lactose intolerance or CMA.^37^ Nowadays, more than 60% of the world population has lactose intolerance, and its occurrence generally increases with age.^38^ Therefore, lactose intolerance and CMA treatment involve restricting dairy products in the diet, which may compromise calcium intake in childhood without supplementation.^37,39^ Furthermore, misdiagnoses and the dissemination of lactose-free diets in healthy children can contribute to the low percentage of dairy products in their diets with a consequent reduction in calcium consumption.

The differences in calcium consumption between the private and public daycare centers demonstrate that the school team can dictate a greater or lesser inclusion of dairy products and calcium-rich foods in the child's diet. In addition, advertisements for foods low in calcium, such as soft drinks and ultraprocessed foods in school canteens, can induce an increasingly less nutritious and healthy diet.^29,40^ For example, soft drinks have a high concentration of caffeine and phosphate, increasing calciuria and decreasing bone mass gain.^41,42^

For an adequate intestinal absorption of calcium, optimal levels of vitamin D are required. Despite its endogenous production by skin exposure to ultraviolet B sun rays, its dietary intake becomes essential to achieve daily needs in children with insufficient sun exposure. Bueno et al. identified higher inadequate vitamin D intake rates in preschoolers from private and public schools.^13,26,43^ Therefore, the fortification of foods with calcium and vitamin D is an alternative measure adopted to prevent or correct nutritional deficiencies. For example, the food industry of the United States and Canada already produces calcium and vitamin D-fortified foods with good bioavailability, such as apple and orange juices and breakfast cereals.^44,45^ However, these foods are still considered ultraprocessed and may increase the consumption of sugar and food additives during childhood. Therefore, a policy to encourage the intake of fresh and nutrient-rich foods would be ideal for improving the nutritional health of preschool and school children.^46^

Therefore, malnutrition, school nutrition policies, socioeconomic status of families, dissemination of restrictive diets without adequate nutritional support, increased diagnosis of lactose intolerance and CMA in childhood, and exceeding consumption of soft drinks and low calcium food may justify dietary calcium intake below the recommended values for age in preschoolers and schoolchildren.

One of the strengths of this study is the demonstration of the low consumption of dietary calcium in preschoolers and schoolchildren from different regions of Brazil through the compilation of cross-sectional and cohort studies that evaluated calcium, milk, and dairy products intake. Therefore, it is noteworthy that the narrative design of this review limited our ability to draw definite conclusions. Because of that, more investigations are needed to identify risk factors and adopt corrective measures, especially in schoolchildren, to reduce comorbidities during adulthood.

The inadequacy of dietary calcium intake seems to be prevalent in Brazil during childhood, mainly in schoolchildren. Therefore, the evaluation of milk and dairy products intake must be a point to be taken care of to carry out corrective measures in this age group.

## References

[1] Michigami T (2019). Skeletal mineralization: mechanisms and diseases. Ann Pediatr Endocrinol Metab.

[2] Rizzoli R, Bianchi ML, Garabédian M, McKay HA, Moreno LA (2010). Maximizing bone mineral mass gain during growth for the prevention of fractures in the adolescents and the elderly. Bone.

[3] Body JJ, Bergmann P, Boonen S, Devogelaer JP, Gielen E, Goemaere S (2012). Extraskeletal benefits and risks of calcium, vitamin D and anti-osteoporosis medications. Osteoporos Int.

[4] Das S, Choudhuri D (2021). Role of dietary calcium and its possible mechanism against metabolic disorders: a concise review. J Food Biochem.

[5] Sociedade Brasileira de Pediatria (2018). Departamento de Nutrologia. Manual de alimentação: orientações para alimentação do lactente ao adolescente, na escola, na gestante, na prevenção de doenças e segurança alimentar.

[6] Brazil - Ministério da Economia (2019). Pesquisa de orçamentos familiares 2017-2018: primeiros resultados.

[7] Dror DK, Allen LH (2014). Dairy product intake in children and adolescents in developed countries: trends, nutritional contribution, and a review of association with health outcomes. Nutr Rev.

[8] Ouzzani M, Hammady H, Fedorowicz Z, Elmagarmid A (2016). Rayyan-a web and mobile app for systematic reviews. Syst Rev.

[9] Leroux I, Ferreira A, Paniz F, Silva F, Luz M, Batista B (2019). Brazilian preschool children attending day care centers show an inadequate T micronutrient intake through 24-h duplicate diet. J Trace Elem MedBiol.

[10] Martino H, Ferreira A, Pereira C, Silva R (2010). Anthropometric evaluation and food intake of preschool children at municipal educational centers, in South of Minas Gerais State, Brazil. Cien Saude ColetRevCiêncSaúdeColetiva.

[11] Tavares B, Veiga G, Yuyama L, Bueno M, Fisberg R, Fisberg M (2012). Nutritional status and energy and nutrients intake of children attending day-care centers in the city of Manaus, Amazonas, Brazil: are there differences between public and private day-care centers?. Rev Paul Pediatr.

[12] Bernardi J, Cezaro C, Fisberg R, Fisberg M, Rodrigues G, Vitolo M (2011). Dietary micronutrient intake of preschool children at home and in kindergartens of the municipality of Caxias do Sul (RS), Brazil. Rev Nutr.

[13] Bueno MB, Fisberg RM, Maximino P, Rodrigues GP, Fisberg M (2013). Nutritional risk among Brazilian children 2 to 6 years old: a multicenter study. Nutrition.

[14] Alencar M, Barros S, Borges I, Cavalcante K, Melo M, Nunes I (2016). Adequacies and inadequacies in the anthropometric and dietetic profiles of preschool children. J Hum Growth Dev.

[15] Suhett L, Silveira B, Filgueiras M, Peluzio M, Hermsdorff H, Novaes J (2018). Inverse association of calcium intake with abdominal adiposity and C-reactive protein in Brazilian children. Public Health Nutr.

[16] Scagliusi F, Garcia M, Indiani A, Cardoso M (2011). Relative validity of a food-frequency questionnaire developed to assess food intake of schoolchildren living in the Brazilian Western Amazon. Cad SaúdeSaude PúblicaPublica.

[17] Nascimento K, Silva B (2018). Impact of a nutrition intervention to the dietary practices of students of a private school. Rev Baiana Saúde Pública.

[18] Magalhães EI, Pessoa MC, Franceschini SD, Novaes JF (2017). Dietary calcium intake is inversely associated with blood pressure in Brazilian children. Int J Food Sci Nutr.

[19] Oliveira S, Filha E, Araujo J, Barbosa J, Gaujac D, Santos C, Silva D (2012). Consumption of food groups among children attending the public health system of Aracaju, Northeast Brazil, in Sergipe. Rev Paul Pediatr.

[20] Bortolini G, Vitolo M, Gubert MS, Santos LM (2013). Early cow's milk consumption among Brazilian children: results of a national survey. J Pediatr (Rio J).

[21] Rauber F, Hoffman D, Vitolo M (2014). Diet quality from pre-school to school age in Brazilian children: a 4-year follow-up in a randomised control study. Br J Nutr.

[22] Bielemann R, Vaz J, Domingues M, Matijasevich A, Santos I, Ekelund U (2018). Are consumption of dairy products and physical activity independently related to bone mineral density of 6-year-old children? Longitudinal and cross-sectional analyses in a birth cohort from Brazil. Public Health Nutr.

[23] Martins M, Aires J, Dantas K, Sabino L, Alves M, Ximenes L (2015). Food consumption among families of pre-school children in situation of food (in)security. Cienc y EnfermeriaCiencia y Enfermeria.

[24] Pretto A, Kaufmann C, Dutra G, Albernaz E (2014). Prevalence of factors related to the bone mass formation of children from a cohort in Southern Brazil. Nutr Hosp.

[25] Levy-Costa R, Monteiro C (2004). Cow's milk consumption and childhood anemia in the city of São Paulo, southern Brazil. Rev Saúde Pública.

[26] Bueno AL, Czepielewski MA (2008). The importance for growth of dietary intake of calcium and vitamin D. J Pediatr (Rio J).

[27] Castro M, Verly E, Fisberg M, Fisberg R (2014). Children's nutrient intake variability is affected by age and body weight status according to results from a Brazilian multicenter study. Nutr Res.

[28] Pessoa J, Shlomo L, Longui C, Mendonça B (1997). Bone mineral density: correlation with body weight, height, bone age and insulin-like growth factor. J Pediatr (Rio J).

[29] Greer F, Krebs N (2006). American Academy of Pediatrics Committee on Nutrition. Optimizing bone health and calcium intakes of infants, children, and adolescents. Pediatrics.

[30] Fisberg RM, Marchioni DM, Colucci AC (2009). Assessment of food consumption and nutrient intake in clinical practice. Arq Bras Endocrinol Metab.

[31] Brazil - Ministério da Saúde (2015). Departamento de Atenção Básica. Saúde da Criança - Aleitamento materno e alimentação complementar.

[32] Brazil - Ministério da Economia (2016). Pesquisa Nacional de Saúde do Escolar 2015.

[33] Pereira G, Genaro P, Pinheiro M, Szejnfeld V, Martini L (2009). Dietary calcium – strategies to optimize intake. Rev Bras Reumatol.

[34] Buzinaro EF, Almeida RN, Mazeto GM (2006). Bioavailability of dietary calcium. Arq Bras Endocrinol Metabol.

[35] Siqueira E, Mendes J, Arruda S (2007). Mineral bioavailability in vegetarian and omnivorous meals served in a university restaurant. Rev Nutr.

[36] Mangels AR (2014). Bone nutrients for vegetarians. Am J Clin Nutr.

[37] Heine RG, AlRefaee F, Bachina P, De Leon JC, Geng L, Gong S (2017). Lactose intolerance and gastrointestinal cow's milk allergy in infants and children - common misconceptions revisited. World Allergy Organ J.

[38] Storhaug CL, Fosse SK, Fadnes LT (2017). Country, regional, and global estimates for lactose malabsorption in adults: a systematic review and meta-analysis. Lancet Gastroenterol Hepatol.

[39] Santos GJ, Rocha R, Santana GO (2019). Lactose intolerance: what is a correct management?. Rev Assoc Med Bras.

[40] Martins P, Pimenta A, Martins D (2008). nfancy and adolescence: is the feeding sufficient in calcium and phosphorus?. Rev Med Minas Gerais.

[41] Chen L, Liu R, Zhao Y, Shi Z (2020). High consumption of soft drinks is associated with an increased risk of fracture: a 7-year follow-up study. Nutrients.

[42] Al-Othman A, Al-Musharaf S, Al-Daghri NM, Yakout S, Alkharfy KM, Al-Saleh Y (2012). Tea and coffee consumption in relation to vitamin D and calcium levels in Saudi adolescents. Nutr J.

[43] Ramasamy I (2020). Vitamin D metabolism and guidelines for vitamin d supplementation. Clin Biochem Rev.

[44] Marques M, Marques MM, Xavier ER, Gregório EL (2013). Food fortification: an alternative to meet the needs of micronutrients in the contemporary worldFortificação de alimentos: uma alternativa para suprir as necessidades de micronutrientes no mundo contemporâneo. HU Revista.

[45] Cormick G, Betran AP, Romero IB, Cormick MS, Belizán JM, Bardach A (2021). Effect of calcium fortified foods on health outcomes: a systematic review and meta-analysis. Nutrients.

